# Breaking Bad News to a Prospective Cross-Sectional Sample of Patients’ Relatives in a Nigerian Neurosurgical Service

**DOI:** 10.3389/fneur.2013.00110

**Published:** 2013-08-05

**Authors:** Amos Olufemi Adeleye, Akinola A. Fatiregun

**Affiliations:** ^1^Department of Surgery, Division of Neurological Surgery, College of Medicine, University of Ibadan, Ibadan, Nigeria; ^2^Department of Epidemiology and Medical Statistics, College of Medicine, University of Ibadan, Ibadan, Nigeria

**Keywords:** breaking of bad news, patient’s family’s preference, native Africans

## Abstract

**Objectives:** Breaking of medical bad news is anecdotally deemed culturally unacceptable, even intolerable, to native Africans. We explored this hypothesis among a cohort of relatives of patients who had difficult neurosurgical diagnoses in an indigenous practice.

**Materials and Methods:** A semi-structured, interviewer-administered questionnaire was used in a cross-sectional survey among a consecutive cohort of surrogates/relatives of concerned patients. Their opinion and preferences regarding the full disclosure of the grave neurosurgical diagnoses, and prognoses, of their wards were analyzed.

**Results:** A total of 114 patients’ relatives, 83 (72.8%) females, were sampled. They were mainly young adults, mean age 40.2 (SD 14.2) years; 57% had only basic literacy education; but the majority, 97%, declared themselves to have serious religious commitments. Ninety nine percent of the study participants deemed it desirable that either they or the patients concerned be told the bad news; 80.7% felt that this is best done with both patients and relations in attendance; 3.5% felt only the patients need be told. These preferences are similar to those expressed by the patients themselves in an earlier study. But a nearly significant greater proportion of patients’ relatives (15 vs 5%, *p* = 0.06) would rather be the only ones to be told the patients’ bad news.

**Conclusion:** This data-driven study showed that contrary to anecdotal belief about them, a cohort of native Nigerian-African surrogates of neurosurgical patients was well disposed to receiving, and appeared able to handle well, the full disclosure of difficult medical diagnostic/prognostic information.

## Introduction

Breaking of bad news in medical practice is about truth-telling. Or the full disclosure to the patients and or their surrogates of all information about their medical diagnoses, and prognoses, even if these be deemed to be grave by the attending physicians (1–4). Unlike what obtains in the more open societies of the Western hemisphere of our world, breaking of bad news in medical practice to the patients is still presumed to be unwise and probably counter-productive in native Africa (5). So also in some other racial groups elsewhere including the Italians, the Spanish, Chinese, Japanese, and others in Asia and Eastern Europe (6–8).

More specifically however, the subject of medical breaking of bad news is particularly remarkable in the African context for the dearth of data-driven literature on the issue. We know of only few anecdotal opinions cum occasional commentaries/editorials that are freely available in this respect (5, 9). In an earlier report we showed the findings from our data-driven study on this subject from an indigenous African in-hospital patient population. This was a prospective consecutive cross-sectional survey of native African patients in a neurosurgical practice (10). We had carried out the same survey on the relatives/surrogates of those same patient cohorts. Here now we present our findings of the follow-up study.

It is a prospective cross-sectional survey of the profiles of difficult neurosurgical diagnoses given to patients and their relatives in the principal author’s practice, and the disposition of the relatives to the breaking of the news. We also compared the responses of the relatives so extracted with those of their own wards (the patients) that were earlier analyzed.

## Materials and Methods

This study was a prospective cross-sectional survey spanning a period of one and half years from July 2008 until December 2009. Consenting native Nigerian-African adults who had grave neurosurgical diagnoses given their respective patient relations were consecutively interviewed. Our findings from the study of the patients’ own responses on the same theme was presented in an earlier report (10).

Such life-altering clinical diagnoses are usually given in clear terms (including the vernacular when necessary), to patients and their most significant relations in the principal author’s neurosurgical practice. Examples include irrecoverable limb paralysis from acute (usually traumatic) or chronic (usually neoplastic/inflammatory) spinal cord disease; irrecoverable neurologic deficits (e.g., bilateral blindness, usually nil light perception) from delayed presentation of brain tumors (11); or a diagnosis of malignant brain tumor with expected limited length of survival following presentation/treatment.

The data-collection tool was a semi-structured, pre-tested, interviewer-administered questionnaire. This was used to explore, one-on-one with each patient’s relative, their disposition to, and their opinion about the propriety or otherwise of the medical bad news they had received. The interview usually took place within the first few days (usually less than a week) of admission and commencement of medical investigation/therapy. Recruitment of study participants and data gathering were coordinated by senior resident doctors in our unit who were not privy to the objectives of the study.

Information included in this questionnaire (Appendix) first of all established the brief demographics of each patient’s relative, hereafter referred to as “study participant(s)”; and the specific clinical diagnoses of the patients for whom they cared. The next domain on the questionnaire established that study participants had been informed of the life-altering clinical diagnoses of their wards’, and then tested their understanding of the full implications of these diagnoses to their wards’ future life courses. Next, the study participants’ disposition to the fact of being told the bad news was explored. Specifically, they were asked whom they would like the news be told, whether the patients and or their relatives (and why so, if only the relatives), and how soon after the diagnosis. Finally, they were asked the question “Or would you rather that you were not told, you or the patient, about this diagnosis?”

### Statistical analysis

The data from the questionnaire were analyzed with the Statistical Package for the Social Sciences (SPSS) version 15 (SPSS Inc., Chicago, IL, USA). Descriptive analysis is presented in sizes (frequencies and means ± standard deviations) and proportions (percentages), and tabular forms. Statistical tests of association were then performed comparing these responses from the study participants with those earlier obtained from the patients for whom these study participants were relations. Continuous variables were tested with the Student’s *t*-test, categorical ones with the chi-square test with alpha value ≤0.05 deemed statistically significant.

## Results

There were 114 patient relations recruited in the study period. The neurosurgical diagnoses associated with grim prognoses given their wards (the patients for whom they cared) are shown in Table 1. These include complete motor with or without sensory paralysis of either all the four limbs, or the lower limbs only, from spinal trauma or other inflammatory/infective spinal lesions in about 80%; and in the rest, permanent neurological deficits (e.g., severe visual impairment, nil light perception) from benign brain tumors, or the diagnosis of malignant brain tumors like glioblastoma, brain stem glioma, and so on.

**Table 1:** **Breaking of bad news in a Nigerian neurosurgical service: comparison of the admission diagnoses of the patients and contemporary patients’ relations surveyed**.

Admission diagnosis	Patients	Relations	*p*-Value
	*N* = 109 (%)	*N* = 114 (%)	
Traumatic quadriplegia	47 (43.1)	44 (38.6)	
Traumatic paraplegia	22 (20.2)	28 (24.6)	
Quad/paraplegia (others)*	17 (15.6)	16 (14.0)	
Benign brain tumor	13 (11.9)	19 (16.7)	
Malignant brain tumor	10 (9.2)	7 (6.1)	0.664

*Quad or paraplegia from other causes like neoplasms or inflammatory diseases of the spinal column and cord.

Table 2 shows the demographics of the study participants, juxtaposed for comparison, with those of their contemporary patient cohorts. They comprised mainly of females, 73%. This is significantly different (*p* < 0.001) from the gender distribution of their contemporary patient cohorts who were more males, 68%, than females. The two study populations were similar in demographics in other respects: mainly young adults with average age of about 40 years; with strong Christian/Islamic religious convictions in overwhelming majority; unemployed/students, artisans/traders, and low/mid-level civil servants in up to 90%; and more than halve of them having only modest literacy educational attainment.

**Table 2:** **Breaking of bad news in a Nigerian neurosurgical service: patients’ and relations’ characteristics**.

Characteristics	Patients	Relations	*p*-value
	*N* = 109 (%)	*N* = 114 (%)	
Mean age in years (SD)	40.2 (14.2)	40.5 (13.6)	0.650
Age range in years	18–78	17–83	
**GENDER**			
Male	74 (67.9)	31 (27.2)	
Female	35 (32.1)	83 (72.8)	<0.001
**EDUCATION**			
Primary school or less	25 (23.0)	39 (34.2)	
Secondary (high) school	36 (33.0)	26 (22.8)	
Higher education	48 (44.0)	49 (43.0)	0.102
**OCCUPATION**			
Trading/artisan	51 (46.8)	59 (51.8)	
Low-mid level civil servants	35 (32.1)	26 (22.8)	
Unemployed/students	15 (13.8)	24 (21.1)	
Professionals	8 (7.3)	5 (4.4)	0.206
**RELIGION**			
Christianity	78 (71.6)	82 (71.9)	
Islam	31 (28.4)	32 (28.1)	0.95
**RELIGIOUS FERVENCY**			
Not religious	1 (0.9)	3 (2.6)	
Religious	55 (50.5)	58 (50.9)	
Very religious	53 (48.6)	53 (46.5)	0.616

*Shows the comparison of characteristics of patients and their relations who participated in the study. Apart from gender which showed that significantly most relations 72.3% were women compared with patients who were women (32.1%), there were no significant differences between the other attributes of patients and relations among the study participants*.

All the study participants admitted that they had been told their wards’ diagnoses and also about the possibilities of the not-too-favorable nature of their prognoses. Ninety four of them (82.5%) offered that they fully understood the diagnoses; and this level of understanding of the diagnoses and their respective challenging prognostic implications as displayed by each subject was adjudged to be at least reasonable in 57 (50.9%), and fully so in 45 (40.2%). Those patient relations who showed apparent lack of insight about the possible grave nature of their respective ward’s diagnosis and prognosis thereafter had these reiterated to them. And in the light of their understanding of the seriousness of these diagnoses, as many as 109 (>95%) of the subjects averred that this portended significant life-course-altering change to the future of their wards.

With only one exception, nearly all, 113 (>99%), of the study participants deemed it desirable, and that as soon as possible, that either they or the patients concerned be in the know of the medical bad news, Table 3. This included 92 (80.7%) who felt that this is best done with both patients and relations in attendance; and 4 (3.5%) who felt only the patients need be told. These preferences are similar to those expressed by the patients themselves. But a nearly significant greater proportion of these patients’ relatives (15 vs 5%, *p* = 0.06) would rather be the only ones to be told the patients’ bad news. More than three quarters of these feared the patient would be devastated by the news. The rest of the relatives who would not have the patient be told the bad news felt it would do the patients no good.

**Table 3:** **Breaking of bad news in a Nigerian neurosurgical service patients and relations opinion and preference**.

Opinion and preference	Patients	Relations	*p*-value
	*N* (%)	*N* (%)	
To whom the news be broken	*n* = 109	*n* = 114	
Patient only	7 (6.4)	4 (3.5)	
Patient and relation(s)	96 (88.1)	92 (80.7)	
Only the relation(s)	5 (4.6)	17 (14.9)	
Prefer not to know	1 (0.9)	1 (0.9)	0.06
When to break the news	*n* = 105*	*n* = 113*	
As soon as possible after diagnosis/admission	89 (84.8)	97 (85.8)	
Much later during the admission	11 (10.1)	11 (9.7)	
After the initial hospitalization	5 (4.8)	5 (4.4)	0.98
Lifestyle changes	*n* = 109	*n* = 111**	
Completely new beginning	24 (21.8)	21 (18.9)	
A great change	50 (45.5)	58 (52.3)	
Moderate change	33 (30.0)	30 (27.0)	
None	3 (2.7)	2 (1.8)	0.749

*Four subjects among patients and one among relations were undecided.

**Three subjects among patient relations undecided.

*This table is a comparison of relations’ opinion and preference on breaking of bad news with that of a contemporary patient population. Although a higher proportion of relations opined that relations only (14.9%) should be informed of the bad news compared to patients’ (4.6%), this did not reach statistical significance. There was also no significant difference in the preference of patients and relations regarding when to break the bad news*.

## Discussion

This is a follow-up study to the earlier one reported by us on the subject of the breaking of medical bad news to native African patients in a Nigerian neurosurgical service. In that earlier report we showed that unlike what is commonly believed about them that native Africans may no longer be really averse to breaking bad news. That document appeared to be the first data-driven one on this subject in any indigenous African patient population (10). Specifically in that report not only did as much as 80% of the study population feel being given the full disclosure of their grave medical news would do them some measurable good, and not any harm as commonly feared; only about 6% of them would rather not be told. It is just that the majority would rather the difficult news be told them in the company of their surrogates, relatives or other significant others.

This companion study explored the same theme with a prospective contemporary consecutive cohort of relatives of those same patients in the same Nigerian neurosurgical service. These cohorts had been with their wards when the news of their grave neurosurgical diagnoses was delivered unto them. The demographics of these relatives were essentially identical to those of the patients except in one respect, the gender distribution. Males (68%) predominate amongst the patient population, male:female ratio 2:1, whereas the overwhelming majority of the carers of the patients were females, 73%, male:female ratio 1:3. This may be partly because much of the grave neurosurgical disease profiled in this study is due to neurotrauma and the young, probably more adventurous males are known to be more prone to injury than their female counterparts in this part of the world (12). It may also be another affirmation of that apparent trend where females are the more likely of the two genders to be seen available caring for their wards in hospital settings (13).

But more specifically speaking, this study also revealed that this cohort of patients’ relatives, born and bred, and living in native Africa, is actually not unfavorably disposed to having medical bad news be broken to them and to the patients for whom they care (14, 15). And although a sizeable but not statistically significant (*p* = 0.06) proportion of the relatives would like to shield their patient wards, against the patients’ own wish, from the bad news (6, 9, 14, 16–18), there is at least some congruence of opinions between the two groups. And that is that the medical practitioners do not treat them with any condescending paternalism by withholding information, however grave it be deemed, from them. They would rather be told the whole truth, bad news it may be; only that it be done not to the patients alone, or the relatives alone, but with both patients and relatives in attendance (10, 15, 19–21). That no news is actually possibly worse than bad news (1). In the earlier report, a significant proportion of the patients themselves actually felt that being made aware of their true medical prognoses would serve them well in making pragmatic projections for the next phases of their lives (10).

This subject of breaking bad news and the disposition of a racial population to it is of course best explored with large community surveys. Therefore, the findings of this kind of single-institution, single-practice study are obviously probably only to be received with caution. We admit this fact as a necessary limitation of this study. Hence its findings are not to be viewed as being totally representative of the population that has been so probably highly selectively sampled.

All said, it appears from this study, at least, that what had hitherto been believed about certain traditional racial groups, including native Africans, regarding breaking bad news may need rethinking, and new clarifications (19, 21–23). It is hoped that further geographically appropriate, and also hopefully large population surveys, would soon come to light to help in this regard.

## Conclusion

This study is a prospective cross-sectional survey of the disposition to receiving medical bad news of a consecutive cohort of relatives of patients with difficult diagnoses and grave prognoses in a Nigerian neurosurgical practice. Contrary to the general perceptions concerning this subject in native Africans, majority of the study participants deemed it desirable that they and their wards (the patients) be told the whole truth. These study participants and their contemporary patient cohorts only feel that such grave news is best delivered not to the patients alone, nor to the relations alone, but to both of them, the one in the company of the other.

## Conflict of Interest Statement

The authors declare that the research was conducted in the absence of any commercial or financial relationships that could be construed as a potential conflict of interest.

## Appendix

### The Research Tool

BREAKING BAD NEWS: Care giver/Significant other

Most significant other: Father/Mother/Wife/Husband/Child(ren)/Others (state please)

Gender Age Education: Primary/Secondary/Tertiary Occupation

Religion very religious/religious/not religious

Address/phone

### Clinical Diagnosis

1. Have you been fully informed about your relation’s diagnosis? Yes/No
2. By whom? Managing physicians/Relations/Research Team
3. Do you understand the full implications of this diagnosis and the lifestyle changes these portend in your relation (the patient)? Yes/No
4. Please explain these (rating of the level of understanding displayed) Fully/Partially/Not at all
5. What degree of change in the patient’s lifestyles do you think this diagnosis portends? Completely new beginning/a great change/moderate change/none
6. Given your understanding now, to whom would you prefer the report of the diagnosis be told? Patient only/Relations only/Patient and relations
7. If relations only, why so? Patient would be devastated/Does not serve any good purpose for patient to know
8. How soon after the admission/diagnosis do you like to be told? As soon as possible/Much later/After the initial hospitalization
9. Or would you rather that you were not told, you or the patient, about this diagnosis? Yes/No
